# A Radio Channel Model for D2D Communications Blocked by Single Trees in Forest Environments

**DOI:** 10.3390/s19214606

**Published:** 2019-10-23

**Authors:** Imanol Picallo, Hicham Klaina, Peio Lopez-Iturri, Erik Aguirre, Mikel Celaya-Echarri, Leyre Azpilicueta, Alejandro Eguizábal, Francisco Falcone, Ana Alejos

**Affiliations:** 1 Department of Electric, Electronic and Communication Engineering, Public University of Navarre, 31006 Pamplona, Navarra, Spainpeio.lopez@unavarra.es (P.L.-I.); erik.aguirre@unavarra.es (E.A.); alex.egui.2@gmail.com (A.E.); francisco.falcone@unavarra.es (F.F.); 2 Department of Signal theory and Communications, University of Vigo, 36310 Vigo, Spain; hklaina@uvigo.es; 3 Institute for Smart Cities, Public University of Navarre, 31006 Pamplona, Navarra, Spain; 4 School of Engineering and Sciences, Tecnologico de Monterrey, Monterrey 64849, Mexico; mikelcelaya@gmail.com (M.C.-E.); leyre.azpilicueta@tec.mx (L.A.)

**Keywords:** device-to-device, internet of things, wireless sensor networks, vegetation, ray launching, 5G, radio channel model, scattering, log-distance

## Abstract

In this paper we consider the D2D (Device-to-Device) communication taking place between Wireless Sensor Networks (WSN) elements operating in vegetation environments in order to achieve the radio channel characterization at 2.4 GHz, focusing on the radio links blocked by oak and pine trees modelled from specimens found in a real recreation area located within forest environments. In order to fit and validate a radio channel model for this type of scenarios, both measurements and simulations by means of an in-house developed 3D Ray Launching algorithm have been performed, offering as outcomes the path loss and multipath information of the scenarios under study for forest immersed isolated trees and non-isolated trees. The specific forests, composed of thick in-leaf trees, are called Orgi Forest and Chandebrito, located respectively in Navarre and Galicia, Spain. A geometrical and dielectric model of the trees were created and introduced in the simulation software. We concluded that the scattering produced by the tree can be divided into two zones with different dominant propagation mechanisms: an obstructed line of sight (OLoS) zone far from the tree fitting a log-distance model, and a diffraction zone around the edge of the tree. 2D planes of delay spread value are also presented which similarly reflects the proposed two-zone model.

## 1. Introduction

In the last few decades, Wireless Sensor Networks have become indubitably one of the most important technologies for both development and investigation in various fields as health care [1], agriculture [2], domestic monitoring [3], and means of land transport [4], among others. However, although in most of applications, numerous sensor nodes are distributed around a wide geographical area while the reliability of the wireless communication in terms of network coverage is a crucial requirement, some environments can be tough and make it hard to achieve the desired communication between nodes; thus resulting in a non-responsive application. Inhomogeneous vegetation environment is one of the most common cases that cause wireless communication complexities. Although WSN are expected to automatically monitor the ecological evolution and wildfire habits in forests [5,6], as over 400,000 wildfires have occurred in Spain over the last 30 years [7] also many rescue interventions are carried out to retrieve people lost in the woods using mobile wireless communication, inhomogeneous vegetation environments have the special feature of acting as scatterers of electromagnetic waves. The signal scattering is translated into an excess of attenuation which can limit the performance of the Device-to-Device (D2D) communications envisaged at low power, high data rates and low latency expected for the upcoming 5G mobile wireless communications. D2D is expected to be used in almost every environment and scenario, and very especially in smart cells. The reduced coverage area and need for high data rate performance of this type of cell deployment can be limited by the attenuation due to vegetation.

In fact, the appearance of the trees in the path of the communication link between the transmitter and the receiver has significant effects on the quality of the received signal. This is because a forest is characterized by vegetation of different canopies and components with different physical natures in terms of trees height, leaves pattern and thickness, trunk sizes and number of branches, which determine the rate of attenuation of radio waves that propagate through it via scattering, absorption, refraction and diffraction of the waves. The signal scattering is translated into an excess of attenuation which can limit the performance of the Internet of Things (IoT) envisaged at high data rates and low latency expected for the upcoming 5G mobile wireless communications. In this context, radio planning tasks become necessary in order to assess the validity of future D2D communications operating in vegetation environments.

For that purpose, path loss models for scenarios with vegetation play a key role since they provide RF power estimations that allow an optimized design and performance of the wireless network [8]. Moreover, a path loss model may contribute to the evaluation of the maximum effective distance between adjacent terminals and hence to the estimation of the number of sensors needed to cover a certain area. Finally, the signal strength loss is related to the quality of service (QoS), causing unreliable communication between nodes that will increase both the number of data packet retransmissions and the power consumption of the nodes, causing radio link failure in last term. Therefore, there is a need for reliable through-vegetation radio channel modelling for vegetation environments, which will assess the propagation behavior in terms of both path loss and multipath propagation.

Since the 1960s, a significant amount of work has been done to investigate the characterization of the radio channel blocked by vegetation elements [9,10], which proposed different analytical and empirical models to estimate the power attenuation or level excess loss introduced by a signal blockage due to vegetation obstacles, mainly trees [9,10,11,12,13,14,15,16,17,18,19,20,21,22,23]. Among the conventional forest models, we can mention the COST 235 [11] and the Weissberger [12]. These models were developed to model the excess attenuation encountered in a forest beyond that predicted by either free-space or two-ray propagation. Moreover, the path loss was empirically modeled in [11,13,14,15]. In [16,17,18], path loss models for a specific 1.9 GHz radio frequency are presented. More characteristics such as the shadowing loss [19] and small-scale fading [19,20] for trees as well as tall food grass fields [21] were empirically modeled. In addition, a model for ultra-wideband (UWB) channels is presented in [22].

However, although a significant amount of research has been performed on the empirical propagation loss modeling, it is still a challenge to describe the radio wave propagation within the forest environment accurately. Especially in inhomogeneous vegetation environments, since a forest can be made up of mixed or homogeneous tree types resulting in different effects on radio waves even at the same frequency by the same group of trees, depending on the geometry of the link. A model combining path loss and multipath is rarely considered. 

In this contribution, which is an extension of a conference paper [24], we present a simple model to characterize the attenuation due to the isolated trees in an air-to-air communication channel occurring between a static transmitter and a mobile user which moves linearly toward the tree. The developed channel model also considers parameters due to the multipath presence obtaining the value of the delay spread parameter.

Since an accurate modeling of the propagation of radio waves through tree foliage, generally requires accurate electromagnetic description of the tree geometry, including its branches and leaves, the radio characterization was performed by means of simulations based on 3D Ray Launching software, where the specific material parameters of the vegetation elements are considered, such as dielectric constant and conductivity. A geometrical and dielectric model of the trees were created and introduced in the simulation software for two species: pine and oak. The scenario simulated and measured at 2.4 GHz to determine the effects of signal level blockage consisted of a medium-size single oak tree immersed in a forest. The path loss was estimated as dependent of the radio link range. Simulation and measurement results corroborate the existence of a double zone within the path loss curve due to different dominant propagation mechanisms: scattering (near the tree) and line of sight obstruction (far from the tree). Furthermore, 2D planes of delay spread values obtained by simulation similarly reflect this two-zone propagation model.

The measurements were carried out using devices operating at the band of 2.4 GHz. For the isolated tree case, a specific forest (called Chandebrito, situated in Galicia, Spain) composed of different type of trees was the chosen scenario, since it suffered a fire and some isolated trees can be found there nowadays. On the other hand, the Orgi Forest, located in Navarra, Spain, was the selected scenario for the analysis of non-isolated tree case.

The paper is organized as follows. In Section 2, the radio channel characterization for a D2D communications blocked by an isolated single tree is presented. Both simulations by the in-house developed 3D Ray Launching algorithm and measurements in a real forest environment are shown. In Section 3, the same approach is used for the radio channel characterization when the blocking tree is a non-isolated single tree, i.e., when dense vegetation is present in the surroundings. Section 4, Discussion, closes the paper.

## 2. Isolated Single Tree Radio Channel Characterization

In this section, the radio channel characterization at 2.4 GHz when an isolated single tree is blocking the communication path is presented. First, wireless channel estimation methodology based on deterministic 3D Ray Launching algorithm is described and employed. For that purpose, a simulation scenario is implemented, including dimensions and electromagnetic frequency dispersive properties of the employed materials. Then, measurements in a real scenario have been performed in order to complete the obtained simulation-based radio channel model.

### 2.1. Simulation Software

In order to perform wireless channel estimation, in which multiple elements such as vegetation are considered, different approaches can be employed. These can be based on analytical models, usually employing first degree approximations to simplify them, or empirical/semi-empirical models, which rely on measurement-based regressions.

Theoretical or analytical approaches are based on statistical theory and usually provide accurate propagation phenomena predictions [25,26,27,28]. Two different types of theoretical models can be distinguished in the literature, namely the radiative energy transfer (RET) model [29] and the analytical theory approach by Foldy [30]. These two models have successfully been used to simulate radio wave propagation in vegetation environments but, due to the complexity of the mathematical equations on which they are based, and the difficulty to extract the input parameters of the models, such as density, area or thickness of leaves and branches, they tend to be unaffordable for real-sized scenarios, such as forests [31]. 

On the other hand, empirical techniques are based on extensive measurement campaigns in the considered environment [32,33,34,35,36]. They have successfully characterized path loss attenuation impact in diverse scenarios with inhomogeneous vegetation within them, and at different frequency bands. However, their main drawback is their lack of accuracy when different site-specific environments are analyzed. As an example, the work in [37] verifies that the well-known propagation empirical models can lead to an error percentage of 30% in propagation predicted values for a classic tomato greenhouse. 

To overcome these limitations, ray-tracing based methods have been proposed in the literature. In [38], a propagation model based on uniform theory of diffraction (UTD) has been presented for urban environments, considering the impact of propagation over buildings and the vegetation attenuation and scattering. In [39], another propagation model based on geometrical optics (GO) is presented to assess the scattered field caused by vegetation elements in the radio path. Methods based on GO such as ray tracing or ray launching achieve a good trade-off between simulations accuracy and computational cost [40]. Due to this fact, in this work, an in-house developed 3D Ray Launching (3D-RL) algorithm has been used to characterize inhomogeneous vegetation environments. The 3D-RL model is a deterministic technique based on GO and UTD and it is divided in three main steps:

1. The first step consists in the design and creation of a realistic scenario, considering all the obstacles and scatterers within it. All the geometries and dimensions of all the objects within the scenario are taken into account, which lead to a realistic scenario which consider every detail of the environment.
2. The second step is the simulation procedure, in which the physical wavefront is approximated by a set of rays, which travel from the emitter in the same direction as the corresponding wave vector. The rays are launched following a solid angle, with angular resolution in both horizontal and vertical planes. These rays interact with the surrounding media following Fresnel equations, considering the frequency dispersive characteristics of the dielectric constant and conductivity of the corresponding materials of all the elements within the implemented scenario.
3. The last step deals with data processing from the complete 3D simulation volume, in order to extract power characterization as well as time domain characterization of the corresponding system within the simulation scenario.

The ad-hoc 3D ray launching algorithm has been described in detail in [40] and validation in complex environments including vegetation has been described in [41]. In the algorithm, the consideration that the vegetation medium is homogeneous has been assumed, thus it has been treated as an isotropic dielectric material with constant permittivity and conductivity. The 3D RL algorithm considers ray/object interaction in terms of reflection, refraction and diffraction. In particular, diffraction phenomena can be optionally activated in the simulation parameters, which is the case of the simulation results presented in this work.

### 2.2. Model of Tree

**Figure 1:**
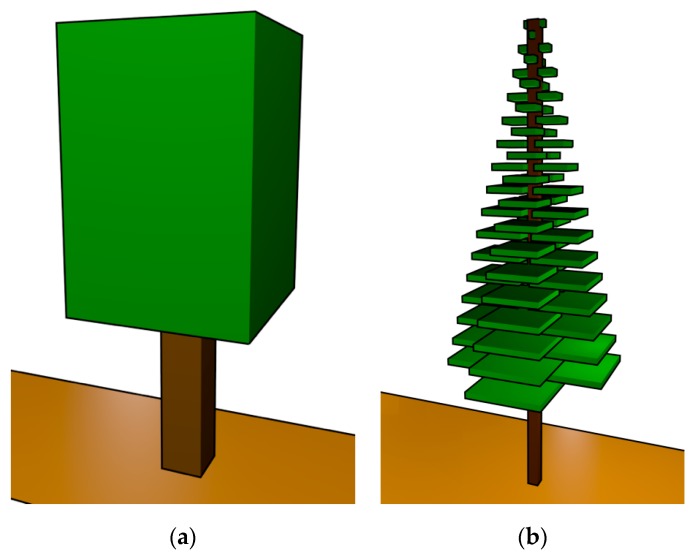
Created tree models for the 3D Ray Launching simulation tool: (**a**) Oak tree; (**b**) Pine tree.

For the presented analysis, the radio propagation through both pines (*Pinus pinaster*) and oaks (*Quercus robur*) has been assessed and specific models have been created in order for them to be considered in the 3D RL simulation code. The structure of oak tree is complex to make a 3D model with RL. Therefore, an approach has been made to respect the main shape of the tree, modelling the mass of leaves as a cube. Usually, three fifths of the height of the tree is mass of leaves and two fifths trunk, although this will depend on the particular oak tree. The oak tree simulation model implemented is composed by a solid trunk and a homogeneous leaf canopy, as depicted in Figure 1a. In the case of the pine simulation model, homogeneous branches with air gaps have been considered. The pine tree branches have a cone-shaped structure with superposition of greater to less length layers starting from the branches closer to the ground (see Figure 1b). The size of the branches depends on the particular pine tree. It is worth noting that both the widths and the heights of the tree models have been parameterized in order to create a computational tree model as closer to the real trees. Moreover, the forest could have a strong influence on the propagation of radio waves. The substrates generally are basic, and rich in acids. In addition, they are usually wet and with accumulation of little leaves and flora remains. Therefore, an appropriate ground has been modelled and included for simulations, since it is an inherent part of a real scenario, which also generates reflections on the propagating electromagnetic wave. The implemented oak and pine tree models are depicted in Figure 1, whereas the corresponding simulation parameters are detailed in Table 1, obtained from [42].

### 2.3. Simulation Results

**Figure 2:**
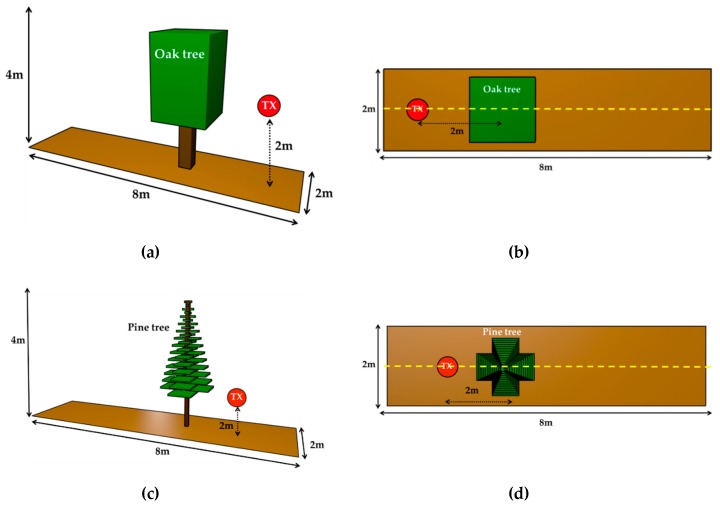
Created scenario for the 3D Ray Launching simulations. (**a**) General view with the oak tree; (**b**) Upper view with the oak tree; (**c**) General view with the pine tree; (**d**) Upper view with the pine tree.

In order to perform wireless channel characterization considering blockage, the implemented simulation scenario considers the location of a static transmitter and a mobile terminal with a thick tree in the propagation path, as depicted in Figure 2. The transmitter source (TX), operating at 2.4 GHz is located at a height of 2 m and is highlighted by a red circle. Material properties and simulation parameters are detailed in Table 1 and Table 2, respectively. The simulation scenario has been implemented with boundary conditions defined by air in order to avoid unwanted reflection components. Note that the parameters regarding the radio signal communications (transmitted power level, antenna type and operation frequency) have been chosen in order to fit the parameters used by the real devices. It is important to mention that the 3D-RL simulation tool has the option to include the effect of diffraction phenomenon. For the current analysis, the diffraction has been activated since it is a relevant phenomenon in the proposed case under study. Simulation results have been obtained for the complete scenario volume. For the sake of clarity, as a specific example, propagation losses for the linear TX-RX radials defined by the yellow dashed lines depicted in Figure 2b,d have been considered for height cut planes of 1, 2 and 3 m.

#### 2.3.1. Path Loss Model

**Figure 3:**
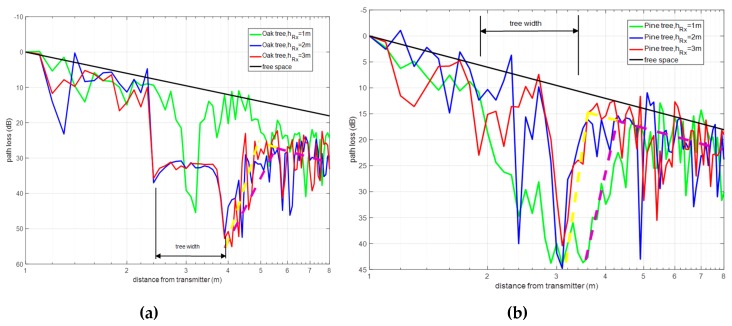
Simulation path loss for a single tree, (**a**) Oak tree; (**b**) Pine tree.

Once the simulation results have been obtained for the scenario, path loss was analyzed considering relative mobile receiver displacement. Results have been obtained considering both oak and pine tree models, for an operating frequency of 2.4 GHz and with increasing distance from the tree location. From the results it can be observed that field scattering owing to the tree location can be divided in two zones: a diffraction dominant zone within the tree vicinity and a free-space zone, depicted in Figure 3. Consequently, at larger distances relative to the tree location, power decay follows free space conditions, owing to dominant line-of-sight (LOS) condition. When the distance from the receiver to the tree location is decreased, the observed response in terms of power decay corresponds to dominant multipath or scattering conditions. In this case, a linear variation of opposite trend to the free-space in received signal is observed, corresponding to a diffraction dominant zone in which relevant tree blockage attenuation recovers, subsequently following a free-space component trend.

The results obtained follow a similar trend as those described in [14], in which a scenario with low elevation is presented. In that case, the propagation path corresponds to an air-to-ground radio link with blockage owing to an isolated tree. The transmitter is located over both the tree and a ground mobile receiver, considering several tree species at frequencies within the X band (8–12 GHz) and Ku band (12–18 GHz). The results presented in [14] identified an OLoS region as well as two different scattering zones: a diffuse scattering-dominant region within the tree trunk vicinity, in which only a statistical distribution function model was followed by the signal level; and a colliding region in which prevalence of tree crown diffraction is observed, modelled considering knife-edge diffraction loss with a correction of the tree height. Beyond this second scattering zone, the signal recovers the power decay corresponding to the OLoS model.

In the presented model, the region within the vicinity of the tree canopy corresponding to diffuse scattering phenomena has not been clearly identified. For the oak tree, this may be due to the absence of air gaps in the simulation model adopted for the mass of leaves which would turn the propagation media into a multi dispersive material. In the case of the pine tree, the simulation model already includes a sufficient volume of air gaps offering a more realistic approach. An experimentally derived model based on anechoic chamber measurements is presented in [14]. For actual trees the mass of leaves is not homogeneous as in the simulation. A diffuse scattering is created, given by random nature of fading owing to interaction with the leaves. It is worth noting that signal attenuation as observed in Figure 3a, corresponding to the oak tree case, strongly decreases, which is in principle given by the plausible existence of a diffuse scattering zone. 

Experimental measurements later shown in Section 2.4 have corroborated this fact. Figure 3b depicts the results considering the pine, which are similar with lower definition, given mainly by lower attenuation considering air gaps in the pine model vs. the homogeneous model employed in the case of the oak tree.

#### 2.3.2. Delay Spread

**Figure 4:**
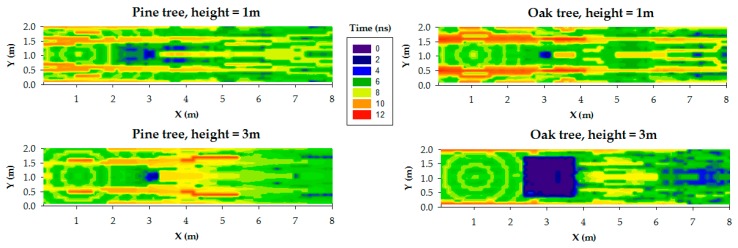
Simulation results of the Delay Spread, for both pine tree (**left**) and oak tree (**right**).

One of the characteristics of the deterministic 3D RL algorithm is the capability to provide multipath characterization, a feature seldom considered in previous models. In Figure 4 it is presented the simulation results of the Delay Spread (DS) as seen at different 2D height planes, for the single tree scenario simulated in Section 3 with the pine and oak tree models. A 2D plane of DS indicates the values of DS detected at each point of a plane located at a specific height, assuming a static transmitter positioned as in the simulations of Section 3, at a height of 2m. DS values depend on the tree species, the distance of the observation point to the transmitter, the percentage of signal blockage, and the observation 2D height plane. A range between 2 and 12 ns was obtained for the simulated scenarios. It is important to note that in the presented results, the RF power level estimations within obstacles (trunk and mainly the vegetation part of the oak) have not been represented in order to show clearer the radio propagation zones around the trees.

For instance, at height 1 m, the points of the observation plane located after the tree show low-medium values of DS: 6–8 ns for the pine and 6–10 ns for the oak. This situation is due to the tree trunk, which is the most influent part in signal blockage.

However, for the pine case at 3 m height plane, the signal blockage is weaker due to the many air gaps of the pine tree top. Therefore, while pine tree behaves like a low density medium, the oak tree shows the behavior of a homogeneous medium. Generally, the obtained results are in accordance with [43,44]. Specifically, [43] presents UHF radio propagation estimations through a trunk-dominated area, for distances greater than 100 m. The work presented in [44] is closer to the case analyzed in this paper: the authors compute the RMS delay spread of the measured power delay profiles at 5 GHz band along different trails within different forests. They obtained delay spread mean values between 60 ns and 90 ns depending on the trail (and therefore, the distance). These values agree with our simulation results, which correspond to shorter distances (less than 8 m in any case).

Similarly to the results in [45], it is noticed that, for any 2D height plane, the presence of vegetation contributed to the received signal level enhancement, predominantly in the side- and back-scattering regions, contrasted with the significant attenuation caused by absorption and scattering in the forward-scattering region produced after the tree [45].

Furthermore, delay spread values achieved along a linear path reflect a two-zone model similarly to path loss in Section 2.1: in the diffraction predominant zone, the number of signal components coming from scattering inside the tree is larger, and once mostly-coherently added produce larger values of DS; in the OLoS zone, away from the tree, the number of multipath components decrease considerably.

### 2.4. Experimental Measurements

**Figure 5:**
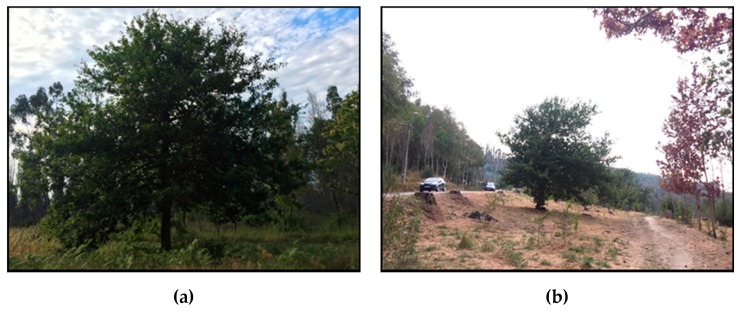
(**a**) Oak tree specimen chosen for experimental measurements. (**b**) Surroundings of the isolated oak tree.

In order to complete the simulation outcomes for path loss, measurements were carried out in a forest and one medium size specimen of oak was chosen. The measurements were carried out using devices operating at the band of 2.4 GHz. The specific forest, composed of different type of trees, is called Chandebrito and is situated in Galicia, Spain. As shown in Figure 5a, the chosen tree’s dimensions are larger in height and width than the model used for simulations: 5.5 m tall and 4.25 m wide. The larger width compensates the lesser homogeneity of the canopy that presents considerably more air gaps than in the simulated model becoming a polidispersive medium [46]. Figure 5b shows the surroundings of the chosen isolated tree, which is at least around 10 m away from any other tree.

The transmitter consisted of a programmable signal generator (WindFreak SynthHD) connected to a directional log-periodic antenna with vertical polarization, model Electro-Metrics EM6952, via a low-loss coaxial cable. The antenna radiation pattern shows 70° of azimuth (E-plane) and 125° of elevation (H-plane). The frequency of the transmitted tone was 2.4 GHz, and its power was 0 dBm. The antenna gain at this frequency is 5.5 dBi.

For acquiring the received signal we used a spectrum analyzer (SA) Rohde Schwarz FSH-6 that registered the RF signal via an antenna identical to the one used in transmission. No amplifiers or filters were used in transmission or reception.

The transmitter antenna was placed on a tripod at a height of 2m, in a fix location at a distance of 3.5 m from the tree trunk base. The receiver antenna was also fixed on the top of a tripod at 2 and 3 meters high, moving along a radial from the tree trunk base to 4.6m apart. Both the transmitter and receiver antennas pointed to the tree canopy.

**Figure 6:**
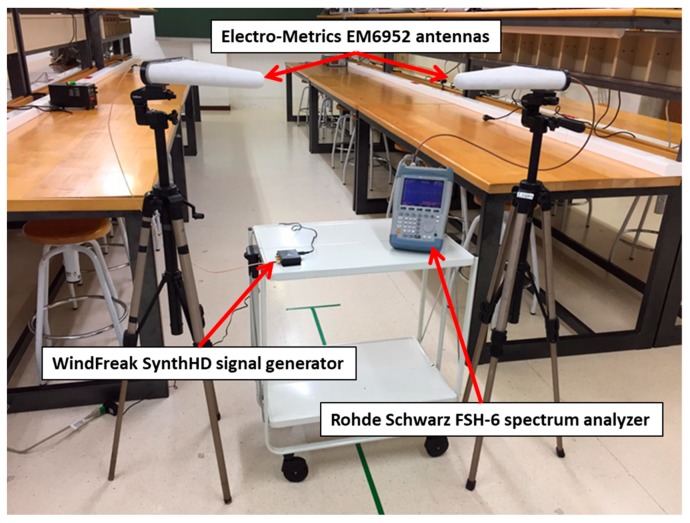
Employed equipment for the measurement campaigns.

The measurement was done moving the tripod with the receiver antenna along a linear path from near (0.25 m) to far (4.6 m) the tree. The initial distance to the tree is 0.25 m. This initial distance was increased in steps of 0.10 m (>λ/4 increments) until the final position at 4.6 m from the tree was reached. At each position of the receiver antenna along the linear path the SA was configured to acquire a data trace of 301 power samples at 2.4 GHz, with a resolution bandwidth of 1 KHz and zero span, in order to average temporal power variations. Measurements were completed for receiver antenna heights of 2 m and 3 m given that in these cases the influence of the tree canopy is what creates the situation of a double propagation zone. A picture of the employed equipment is shown in Figure 6.

#### 2.4.1. Path Loss

The SA measured values were processed. First, an averaging is applied to the 301 power samples composing each trace. Later all the data traces were normalized with respect to the power received at a reference distance of 1m from the transmitter, for each receiver height. In the case of the experimental data, the antenna gains are then compensated. The result is a set of 59 points that determines the signal level loss as a function of the distance between the tree and the receiver. 

**Figure 7:**
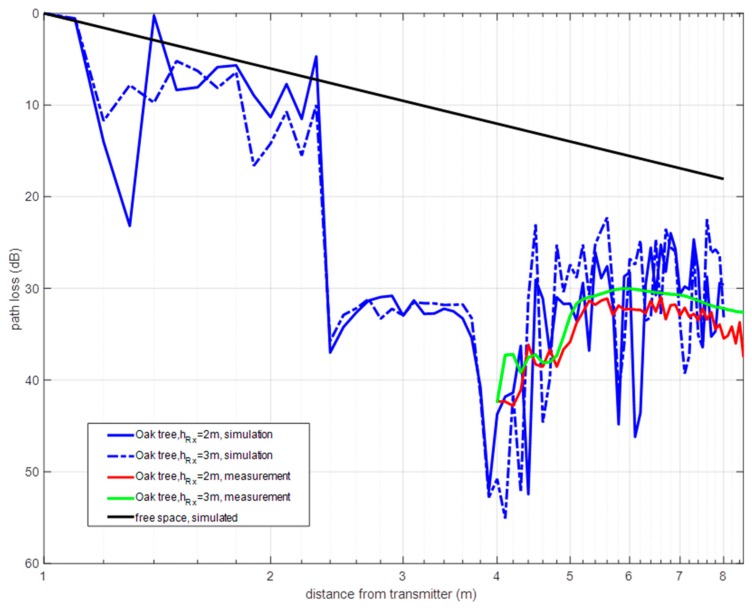
Comparison between simulation and measurement path loss for an isolated oak tree.

The measured path loss is compared to simulated data in Figure 7 (corresponding datasets are provided as supplementary material). Only the portion of the distance axis corresponding to the forwarding zone (after the tree) matches for both datasets. The experimental outcomes corroborate the two-zone propagation model. The difference in dimensions between the virtual tree and the actual tree are primarily reflected in the lower power level received. Moreover, the radiation diagrams, and the power gains of the antennas used differ: omnidirectional in the virtual case and directive in the measurements. These and other factors are responsible for dissimilarities observed between the simulated and the experimental datasets. The not averaged multipath contributions observed in the simulation curves in Figure 7 are likely due to the lack of temporal averaging in the simulated data. The differences in the antenna radiation diagrams of the virtual and measurement cases can also explain the fit discrepancy. The following values of RMSE are observed between the experimental and simulated curves according to the antenna height and the propagation zone:

- For diffuse zone: RMSE = 8.6370 dB at 2 m, and RMSE = 5.2804 dB at 3 m.
- For OLoS zone: RMSE = 5.8596 dB at 2 m, and RMSE = 6.2672 dB at 3 m.

The diffuse scattering zone, located between 4m and around 5.5 m of the virtual distance axis, presents a less pronounced slope for both receiver heights. The slope of the OLoS zone decays faster than in the simulated scenario case. 

Finally, the averaging introduced in the SA acquisition and processing provided lower signal variability than in the simulation results. It is not possible to introduce averaging in the electric field results estimated by the simulation; however, in a simulator, the scenario is static and averaging does not seem necessary. In the measurement scenario, e.g., the wind blowing produces non trivial signal level variations that the averaging filters. The result is that the experimental curves are smoother than the virtual ones. Spatial averaging was not applied; however, it is recommended for windy scenarios or irregularly shaped trees.

**Figure 8:**
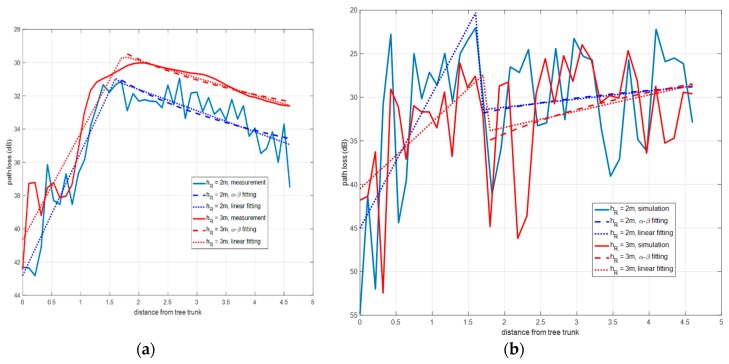
Comparison of path loss curves fitting for an isolated oak tree: (**a**) experimental results and (**b**) simulation-based results.

Typically, the path loss variation as a function of distance is modeled by means of a log-distance or a linear regression. In Figure 8a the results of the model fitting are shown for the measured values, and in Figure 8b for the simulation case. It is noticed that depending upon the radio propagation zone a fit model is more suitable than any other, even despite the receiver height.

The experimental and simulated path loss curves resulted in the following fitting:

1. Diffuse scattering zone: it occurs near the tree, between 0.25 m and 1.6 m from the tree for 2 m height, and up to 1.8 m for 3 m case. In this zone only the fitting could be obtained by linear regression that meets the expression given in Equation (1):
  *PL*(*d*) = *P*_0_ + *n*·*d* (1)
  where *P*_0_ is the reference power loss in dB at the start distance, 0.25 meter from the tree; *d* is the distance in meters between the start distance and receiver; *n* is the factor that determines the power decay rate with the distance. Parameters *P*_0_ and *n* in Equation (1) have been obtained for each one of the two receiver heights.
2. OLoS zone: it occurs for a distance from the tree beyond 1.6 m for 2 m height, and beyond 1.8 m for 3 m case. In this zone the path loss admits a linear regression according to the floating intercept (FI) model Equation (2) applied in [15,20]:
  *PL*(*d*) = α + 10·*β*·log_10_(*d*) (2)
  where *d* is the distance, *β* the line slope, and *α* the floating-intercept in dB. The slopes *β* are −0.818 for 2 m and −0.7047 for 3 m, and the fit errors are 1.053 and 0.2681 dB, respectively. A linear regression was tried with smaller fit errors than the FI model.

As noticed in Figure 8a,b, the division into two propagation zones is more remarkable for the experimental data. The values of the parameters *α*, *β*, *P*_0_ and *n* obtained for each zone and height are summarized in Table 3. The RMS error was used as an estimation of the error of the models.

#### 2.4.2. Signal to Noise Ratio

**Figure 9:**
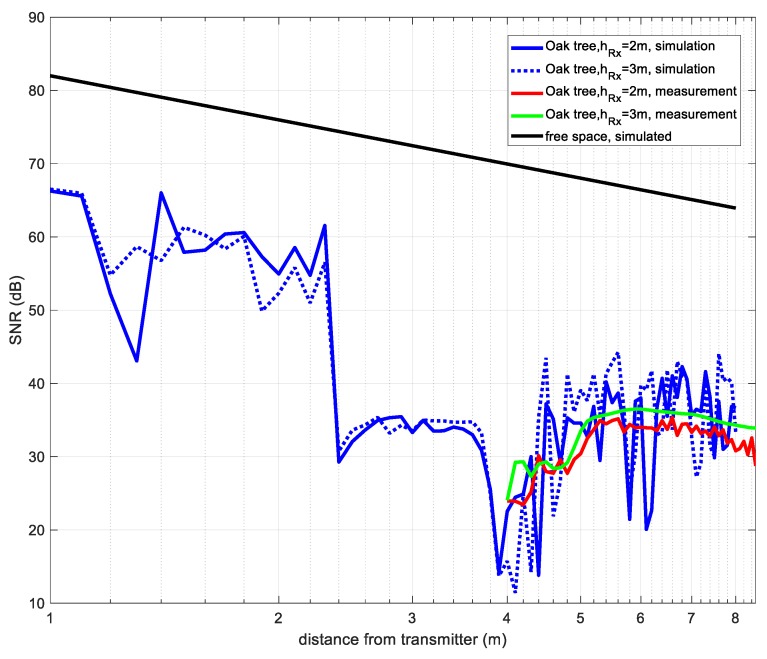
Signal to Noise Ratio (SNR) vs. distance to transmitter for a ZigBee device.

Following the procedure described in [15], it is possible to estimate the coverage distance for an IEEE 802.15.4 connection if the Signal to Noise Ratio (SNR) is obtained as a function of distance. As described in [15], for a 22 byte frame length and a PER of 2%, then a BER < 1.14 × 10^−4^ would be needed. According to [15] and Figure 9 the SNR required is 0 dB approximately. The noise power is measured using the SA, at a central frequency of 2.45 GHz and for a span of 5 MHz that simulates a 5 MHz bandwidth ZigBee channel. The value measured was −82 dBm. 

The resulting SNR is shown in Figure 9. It can be deduced that for a ZigBee system with a threshold of SNR = 0 dB connectivity is possible in the vicinity of the tree. The connectivity is kept for both simulated and experimental cases, even if 2·5.5 dB are subtracted to compensate the antenna gain used in the measurements. This result agrees with [15,20].

This result reinforces the conclusion given in Section 2.3.2 in which it was indicated that the presence of the tree, thanks to the diffraction phenomenon, reinforced the signal reached in certain points around the tree, as also stated in [45]. 

## 3. Non-Isolated Single Tree Radio Channel Characterization

Once the radio channel characterization of an isolated single tree has been performed by means of measurements and simulations, in this section a step towards a more realistic scenario has been taken. Specifically, simulations and measurements of a single tree nearby a dense forest zone with thick in-leaf trees have been performed. This scenario is located in the Orgi Forest. This forest is a 77-hectares millennial forest with a high ecological value which extends into the Ultzama Valley (Navarra). The oak groves populated the Navarra valleys 4000 years ago. Orgi Forest was declared a Natural Recreational Area in 1996, after the intense forest exploitation of wood, pasture for livestock and hunting. This was to encourage the natural regeneration of forest´s flora and fauna, while regulating the use of people.

Orgi has been included in the European Natura 2000 network. Natura 2000 is the largest network of protected areas in the world and it offers protection to most valuable and threatened species in Europe. In the case of Orgi, the oak (Quercus robur) is the main species protected. There are oak trees of two types where many of them are centenarians: the common oak and the American oak.

**Figure 10:**
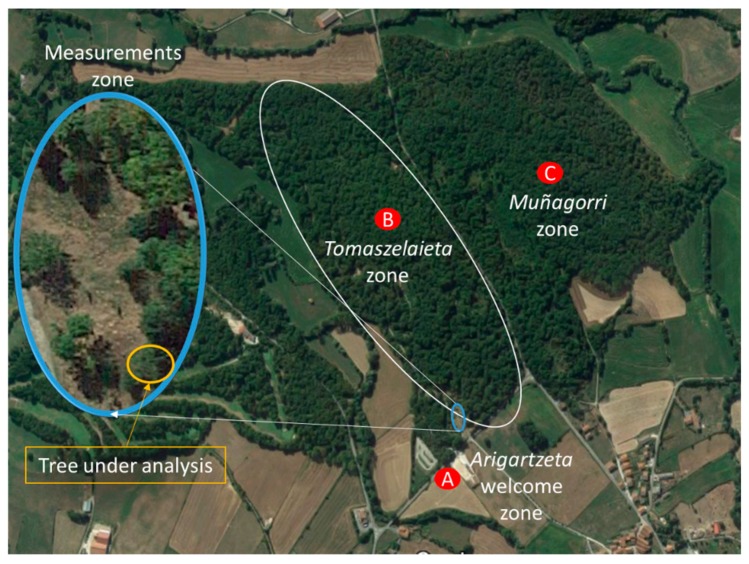
Location of the Orgi Forest and its different zones, including the measurement zone (zoomed). Extracted from Google maps.

**Figure 11:**
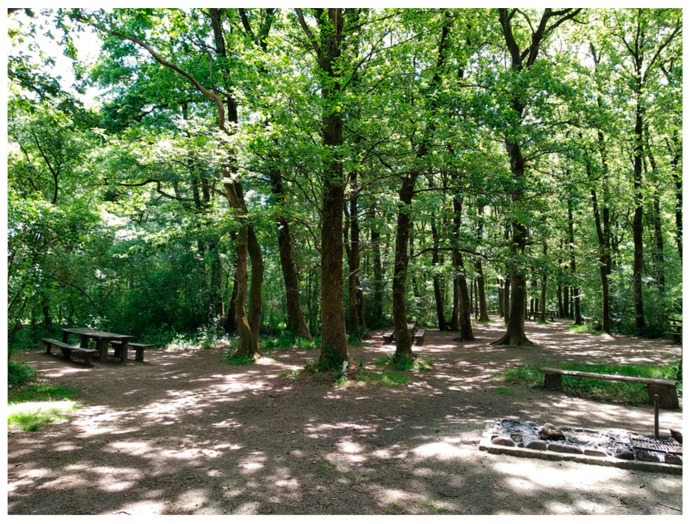
Picture of *Tomaszelaieta* zone within the Orgi Forest.

The forest is organized into three zones (shown in Figure 10). The first called Arigartzeta, is the welcome zone that gives access for the oak grove. It has parking and picnic areas. The second zone is called Tomaszelaieta, and has a large walk area, where the visitor can discover other tree species such as holly and elms as well as some animal species such as birds or amphibians. The third zone is called Muñagorri, which is a conservation area. The public cannot access this last zone due to restrictions in order to facilitate the process of natural regeneration. The measurements have been performed within the accessible Tomaszelaieta zone (see zoomed zone in Figure 10), which is prepared for visitors (see Figure 11).

### 3.1. D Ray Launching Simulations

**Figure 12:**
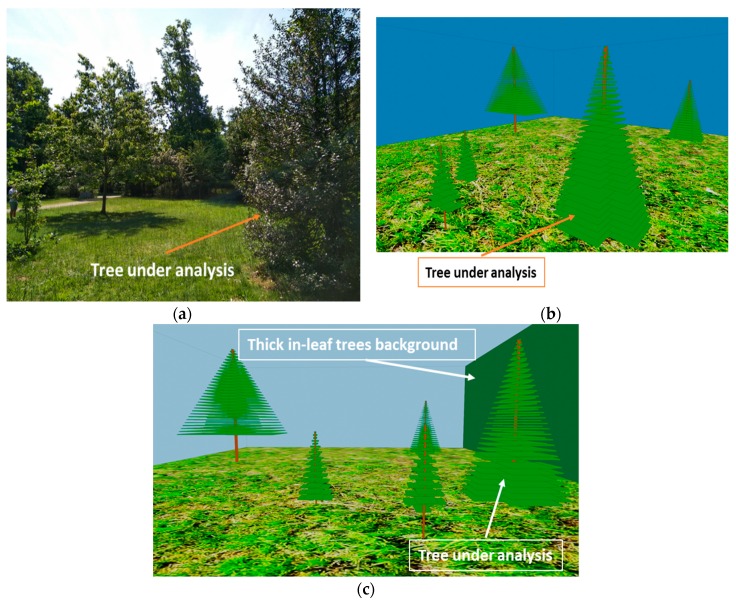
(**a**) Picture of the measurements zone. (**b**) The scenario created for the simulations. (**c**) Detail of the position of the dense vegetation wall.

The specific scenario under analysis is shown in Figure 12a, and it corresponds to the zoomed area shown in Figure 10 (i.e., measurements zone). The scenario has been created for simulations by the 3D Ray Launching tool. All the materials as well as the real distances and sizes of the surrounding trees have been taken into account in order to obtain accurate estimations of the RF power distribution and therefore, the path loss. Both the material properties and simulation parameters are the same as those used for the isolated tree case, and can be seen in Table 1 and Table 2, except the transmitted power, which in this case was 8.35 dBm.

Figure 12b shows the schematic view of the created scenario for simulation purposes. As can be seen, the morphology and size of each of the single trees surrounding the tree under analysis have been taken into account. In the same way, a specific wall has been created in the scenario in order to model the thick in-leaf trees background that exists behind the tree under analysis (the wall in green in Figure 12c). This wall has been defined with the material Foliage (see Table 1), while the rest have been defined as air.

### 3.2. Experimental Analysis

**Figure 13:**
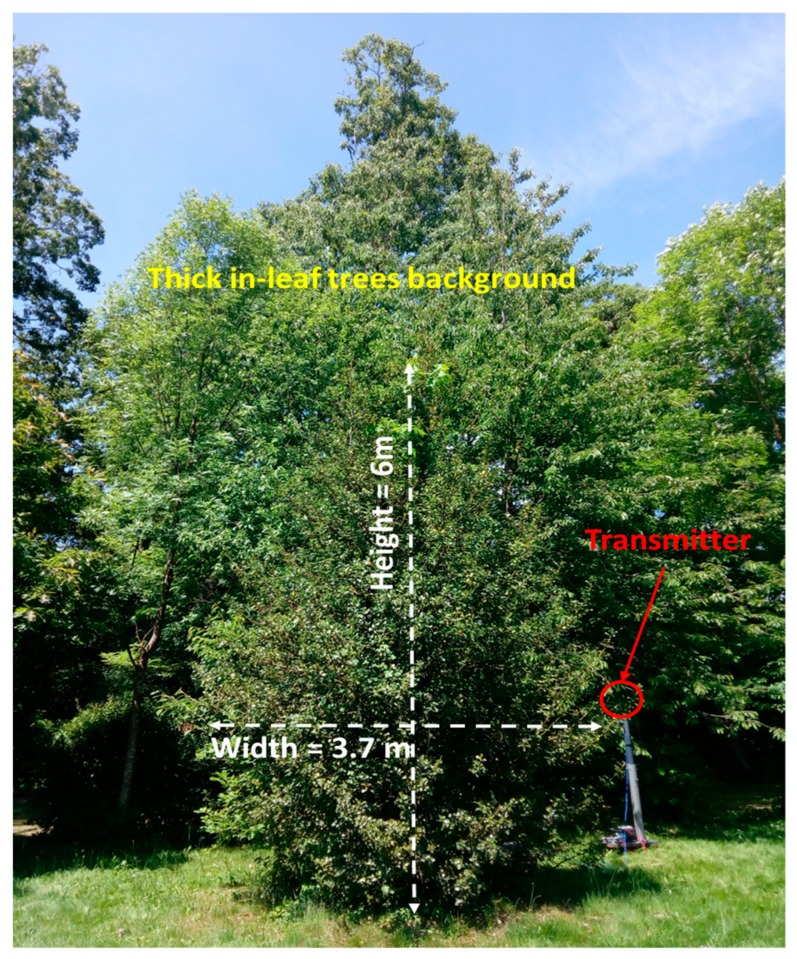
Detail of the Non-Isolated tree under analysis with the dense forest background.

**Figure 14:**
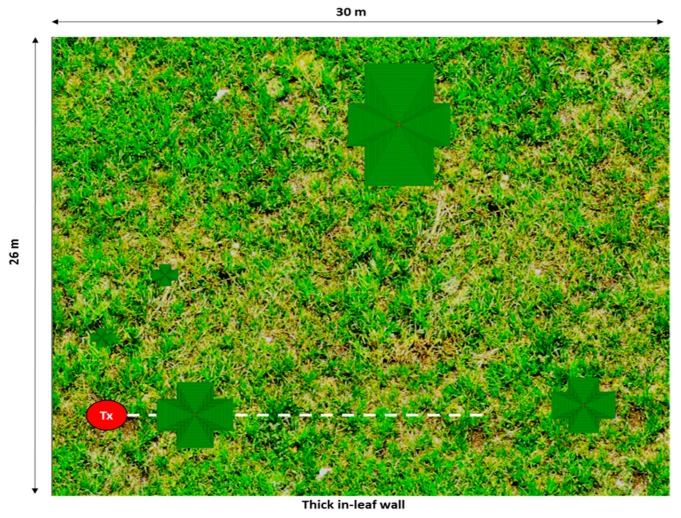
Upper view of the created scenario. The white dashed line represents the linear path where the measurements have been taken.

**Figure 15:**
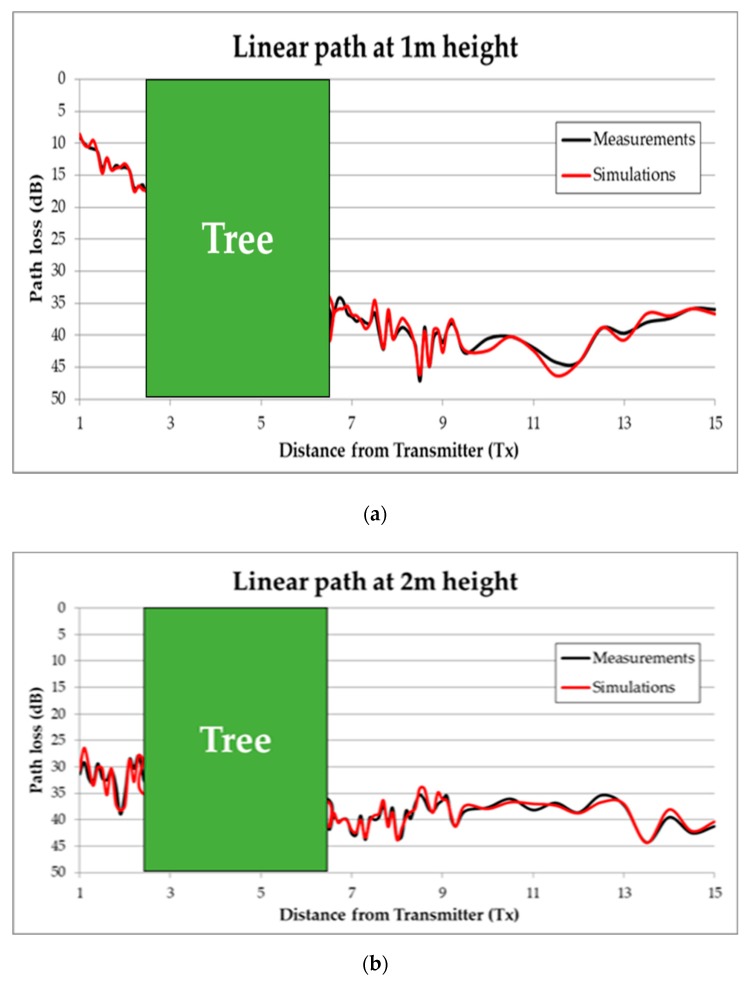
Measured vs. Simulated Path loss for the linear path corresponding to the white dashed line of Figure 14. (**a**) at 1m height; (**b**) at 2 m height.

In this case of non-isolated tree, measurements have been also performed in the real scenario. The transmitter has been placed at 2.5 m distance from the tree under analysis at a height of 1 m, supported by a plastic structure. The tree is 6 m height and 3.7 m of maximum width (see Figure 13). Measurements have been taken in a linear path (represented by a white dashed line in Figure 14) from the transmitter (red dot in Figure 14) to a distance of 15 m. The measured path loss values are depicted in Figure 15, where the simulation results have also been included in order to comparing them. As can be seen, in this non-isolated tree case, the simulator provides very accurate estimations. This good agreement is due to the multipath propagation, which starts gaining relevance because of the dense vegetation present in the nearer area of the tree under analysis.

## 4. Discussion

Observing the values in Table 3, the rate of decay with distance, *n*, appears to be smaller than the value for open scenarios under free-space conditions that is around 2. This indicates the re-radiation effect probably due to the tree. The fitting of the simulation data for the 3 m case exhibits the decay rate value closest to the free-space condition, 1.863.

The parameters values summarized in Table 3, for linear or log-distance models are not in agreement with values reported in [14,15,20]. Reasons for this dissimilarity are found, among others, in the frequency, the scenario, and the radio link setup. Largest RMSE values are observed for the simulation results likely due to the lack of temporal averaging in the simulated data.

In [14], the transmitter antenna points to the top of the tree, and the receiver antenna is pointed upwards, so that the set simulates a transmitter – satellite or helicopter – located over the tree and the receiver. The frequency band analyzed was 8–12 GHz, so that the signal loss is stronger than for the 2.4 GHz: As explained in Section 3, this setup led to three-zone propagation. 

In [15,20], the peer-to-peer transmission is analyzed, without blocking the radio link by a tree, only locating the transmitter on the tree trunk. Using a log-distance model, reported values of the decay rate *n* are larger than 2. In [15,20], a log-distance model is also applied for the radio link blocked by an undefined number of trunks, but not canopies.

In summary, with the exception of [14], most of the literature related to propagation in vegetation media does not reflect a scenario as described herein. Only the RET model [29] could be used to try to replicate the results presented here. However, the difficulty of programming this model, given the high number of parameters to be evaluated, has made this task unbearable. 

For our study of an isolated tree, it is reasonable to consider that any signal fading analysis should not depend on the distribution of the foliage in the species or specimen of the tree considered as would occur for the case of a thin tree. It is for these cases that up-to-date prediction models may not fit the experimental results. For a case of an isolated tree as a vegetation obstacle, some models as ITU-R P.833-7 [13] consider the signal excess loss produced by the diffraction occurring on the obstacle top as well as on the left and right sides of the tree, and these diffractions are modeled according to a knife-edge case.

Our objective is not to offer a model for the components due to the diffraction or scattering phenomena. For modeling those components, a more exhaustive measurement campaign should be carried out, and a wideband channel sounder would be needed to provide power and delay metrics. In our present contribution we provide a path loss model that results sufficient to determine the quality of a D2D link in terms of probability error.

Regarding the results presented in this paper, a diffraction dominant zone in the vicinity of the tree has been identified. Near the tree the channel model corresponds to a multipath response or scattering situation. This region would be dominated by diffraction that is responsible for producing that the large attenuation introduced by the tree blockage starts recovering and continue as an open-space component.

In summary, compared to previous works, in this work experimental results achieved under anechoic chamber have been now demonstrated for outdoors. In addition, it has been also demonstrated that the neighborhood of trees affects the diffuse zone’s positive effect on the received power level. This helps to understand the effect of the single trees on the SNR and the achieved results could find a practical applicability when deploying a network in forest areas.

As previously said, further research is required to deepening in the phenomenon of the propagation in vegetation media, and specifically, more experimental measurements should be carried out at different locations to complete the statistical analysis as well as the results presented in this work. Possible future work would be to analyze the effect of line of trees blocking the wireless propagation. 

It is worth noting that the use of the presented 3D Ray Launching simulation tool gains importance when the scenario under analysis becomes more complex in terms of morphology and the number of elements/obstacles/vegetation present within it, as it can be seen in presented results. Thus, obtaining accurate path loss estimations will lead to optimized radio planning decisions in scenarios with dense vegetation such as parks and forests.

## Figures and Tables

**Table 1:** Material properties for Ray Launching simulations (at 2.4 GHz).

Material	ε_r_	Conductivity (S/m)
Foliage	4.48	0.02
Pine trunk	1.97	0.052
Oak trunk	2.51	0.148
Forest Ground	4.8	0.98

**Table 2:** Ray Launching simulation parameters.

Parameters	Values
Transmitted Power	10 dBm
Operation Frequency	2.4 GHz
Antenna Type/Gain	Monopole/0 dB
Launched rays resolution	1 degree
Permitted maximum rebounds	6
Cuboids size (Mesh resolution)	10 cm × 10 cm × 10 cm
Diffraction phenomenon	Activated

**Table 3:** Path loss fitting parameters for measurement and simulation results.

	Height	Zone	Linear	FI model	RMSE
Measured	2 m	Diffuse	$P_{0}=-42.81$, n=7.412	---	1.491
		OLoS	$P_{0}=-29.07$, n=−1.275	α=−29.18, β=−0.818	0.9687/1.053
	3 m	Diffuse	$P_{0}=-40.64$, n=6.413	---	1.329
		OLoS	$P_{0}=-27.81$, n=−1.037	α=−27.68, β=−0.7047	0.188/0.2681
Simulation	2 m	Diffuse	$P_{0}=-45.02$, n=15.45	---	7.256
		OLoS	$P_{0}=-33$, n=0.9304	α=−33.41, β=0.6963	5.565/5.552
	3 m	Diffuse	$P_{0}=-40.50$, n=7.67	---	5.263
		OLoS	$P_{0}=-37.18$, n=1.863	α=−38.95, β=1.582	6.201/6.096

## Supplementary Materials

The following are available online at https://www.mdpi.com/1424-8220/19/21/4606/s1, Data set DS_sim and DS_meas corresponding to Figure 7: Comparison between simulation and measurement path loss for an isolated oak tree.
